# Final benefit of primary percutaneous coronary intervention for ST-elevation myocardial infarction in older patients: long-term results of a randomised trial

**DOI:** 10.1007/s12471-022-01724-5

**Published:** 2022-09-16

**Authors:** M.-J. de Boer, J. P. Ottervanger, A. W. J. van’t Hof, J. C. A. Hoorntje, H. Suryapranata, F. Zijlstra, Zwolle Myocardial Infarction Study Group

**Affiliations:** 1 Radboud University Medical Centre, Nijmegen, The Netherlands; 2 Isala Hospital, Zwolle, The Netherlands; 3 Maastricht University Medical Centre, Maastricht, The Netherlands; 4 Zuyderland Medical Centre, Heerlen, The Netherlands; 5 Erasmus Medical Centre, Rotterdam, The Netherlands

**Keywords:** Primary percutaneous coronary intervention, Elderly, Survival, Geriatric cardiology

## Abstract

**Background:**

Although the short-term benefit of primary percutaneous coronary intervention (PCI) in elderly patients with ST-elevation myocardial infarction (STEMI) has been demonstrated, the final long-term survival benefit is as yet unknown.

**Aim:**

To assess the final survival benefit of primary PCI as compared to thrombolytic therapy in patients over 75 years of age.

**Methods:**

Patients > 75 years with STEMI were randomised to either primary PCI or thrombolysis. Long-term data on survival were available for all patients.

**Results:**

A total of 46 patients were randomised to primary PCI, 41 to thrombolysis. There were no significant differences in baseline variables. After a maximum of 20 years’ follow-up, all patients had passed away. The patients randomised to thrombolysis died after a mean follow-up duration of 5.2 years (SD 4.9) compared to 6.7 years (SD 4.8) in patients randomised to primary PCI (*p* = 0.15). Thus, the mean final survival benefit of primary PCI was 1.5 years.

**Conclusion:**

The final survival benefit of primary PCI as compared to thrombolysis in elderly patients with STEMI is 1.5 years and their life expectancy increases by 28.8%.

## What’s new?

- A long-term follow-up study of the benefits of primary percutaneous coronary intervention (PCI) in elderly patients has not been performed previously.
- The final survival benefit of primary PCI in the elderly has not been published before.

## Introduction

Primary percutaneous coronary intervention (PCI) for patients with ST-elevation myocardial infarction (STEMI) is nowadays a strong class IA recommendation in the relevant guidelines [1, 2]. Although elderly patients account for a disproportionate number of cases, they have typically been underrepresented in randomised clinical trials. To date, only four studies have specifically randomised only elderly patients with STEMI to either thrombolysis or primary PCI: the Zwolle trial, the Senior PAMI (Primary Angioplasty in Myocardial Infarction) trial, the TRIANA (TRatamiento del Infarto Agudo de miocardio eN Ancianos) trial and a trial from Israel [3, 4] The largest of these trials (Senior PAMI) was never published in full. Moreover, data on long-term effects of primary PCI in elderly patients are lacking, and this may be of particular importance since the life expectancy of older patients is limited often due to comorbidity. We performed a long-term follow-up study of one of the randomised studies to find out the final potential benefit of primary PCI over thrombolysis in elderly patients with STEMI.

## Methods

The long-term results of a randomised trial comparing primary PCI with thrombolytic therapy in older patients with STEMI were analysed. The primary results of the study have been published previously [5].

### Population

From March 1996 until April 1999, 87 patients aged 76 years or older referred directly to our hospital with STEMI and without contraindications for thrombolytic therapy were randomly assigned to treatment with intravenous streptokinase or immediate angiography and, if possible, subsequent angioplasty. Inclusion criteria were as follows: symptoms of STEMI that persisted for more than 30 min accompanied by an elevation of more than 1 mm (0.1 mV) in the ST segment in two or more contiguous electrocardiographic leads; and presentation within 6 h after the onset of symptoms (or between 6 and 24 h if there was evidence of continuing ischaemia). Contraindications to thrombolytic therapy were defined as previous stroke or other known intracranial diseases, recent trauma or surgery, refractory hypertension (systolic > 180 mmHg, diastolic > 110 mmHg), active bleeding or prolonged cardiopulmonary resuscitation. Before randomisation we recorded each patient’s age, gender, Killip class on admission, electrocardiographic site of infarction, history of previous infarction, heart rate, time of onset of symptoms and time of hospital admission. The catheterisation laboratory and the dedicated house staff were available 24 h, 7 days a week. All patients received 450 mg aspirin intravenously, followed by 80 mg aspirin/day orally and intravenous nitroglycerin at a dose designed to maintain a systolic blood pressure of 110 mmHg. Intravenous heparin was given at a dose designed to maintain the activated partial thromboplastin time between two and three times the normal value for at least 2 days. The partial thromboplastin time was measured twice a day. Patients assigned to streptokinase received 1.5 million U intravenously over a period of 1 h. In patients assigned to angioplasty treatment, coronary angiography was performed as soon as possible. Both coronary arteries were visualised; left ventriculography was not performed routinely. Coronary angioplasty was performed at the investigator’s discretion using standard techniques and devices. Patients who received a stent were treated with ticlopidine, 250 mg, twice a day for 2 weeks. All angiograms were reviewed by two experienced investigators not involved in other parts of the study. Flow through the infarct-related vessel (IRV) was scored according to the Thrombolysis In Myocardial Infarction (TIMI) flow grading system, before and after the angioplasty procedure. Agreement on flow and extent of coronary artery disease was reached in all cases. The time from admission to the initiation of therapy was calculated as the time to the start of the streptokinase infusion or the first balloon inflation. Recurrent myocardial infarction was defined as chest pain, changes in the ST‑T segment and a second increase in the creatine kinase level to more than two times the upper limit of normal or an increase of > 200 U/l over the previous value if the level had not dropped below the upper limit of normal. The primary endpoint was the composite of death, reinfarction or stroke at 30 days after randomisation.

### Data collection and follow-up

Data about survival were available for all patients until February 2021. The life expectancy of the study population was compared with that of the general Dutch population (https://opendata.cbs.nl/statline/#/CBS/nl/dataset/37360ned/table?fromstatweb), adjusted for year of inclusion in the study, age and gender. Study approval was obtained from the medical ethics committee of Isala Hospital.

### Statistical analysis

Statistical analysis was performed with the Statistical Package for the Social Sciences version 20.0 (SPSS Inc., Chicago, IL, USA). Continuous data are expressed as mean ± standard deviation and categorical data as a percentage, unless otherwise denoted.

All endpoints were analysed according to the principle of intention-to-treat. A chi-square statistic was calculated to test differences between proportions with calculations of relative risks (RRs) and exact 95% confidence intervals (CIs). Fisher exact test was used if there was an expected cell value of < 5. The Student *t*-test or the Mann-Whitney U test was used to compare continuous values. Survival and survival without recurrent infarction or stroke were calculated by the Kaplan-Meier product-limit method. For all analyses, statistical significance was assumed when the two-tailed probability value was < 0.05.

## Results

### Baseline characteristics

The baseline characteristics are summarised in Tab. 1. The two patient groups were well balanced with regard to the most important determinants of mortality and morbidity. Six patients in the group assigned to PCI and four in the streptokinase group were randomised more than 6 h after symptom onset (*p* = 0.7).

**Table 1:** Clinical characteristics of 87 elderly patients with ST-elevation myocardial infarction randomised to either primary percutaneous coronary infarction (*PCI*) or thrombolysis

Characteristic	Primary PCI *n* = 46	Thrombolysis *n* = 41	*p*-value
Age, years	80 (77–84)	81 (78–84)	0.17
Male gender, *n* (%)	22 (48)	25 (61)	0.31
Diabetes, *n* (%)	11 (24)	7 (17)	0.60
Anterior location, *n* (%)	23 (50)	19 (46)	0.89
Heart rate on admission, beats/min	69 (55–78)	65 (50–80)	0.74
Systolic blood pressure on admission, mmHg	120 (100–145)	120 (100–140)	0.34
Time from onset to admission, min (range)	207 (60–1020)	212 (25–700)	0.62

Of note is that the study was halted prematurely because of ethical issues raised by the medical ethics committee, after the results of the first 80 patients had been analysed.

Of the patients randomised to invasive treatment, 45 underwent coronary angiography, and 41 actually underwent PCI with a procedural success rate of 90% (37 of 41), defined as a residual stenosis of the culprit lesion of < 50% and TIMI 3 flow through the IRV. One patient died before angiography could be performed. Two patients were referred for bypass surgery, and in two patients a conservative treatment was chosen. Four strokes occurred, in one patient in the angioplasty group (1 week after treatment with an intra-aortic balloon pump) and three in the streptokinase group (all on the day of treatment), one of whom died.

### Mortality

After 30 days, three patients (7%) in the angioplasty group had died, compared with nine patients (22%) in the thrombolysis group (*p* = 0.04), resulting in an RR of 4 for death (95% CI: 0.9–24.6) for patients treated with thrombolysis. The composite predefined endpoint of death, recurrent infarction and/or stroke at 30 days occurred in 4 (9%) patients in the angioplasty treated patients versus 12 (29%) in the thrombolysis group (*p* = 0.01).

**Fig. 1:**
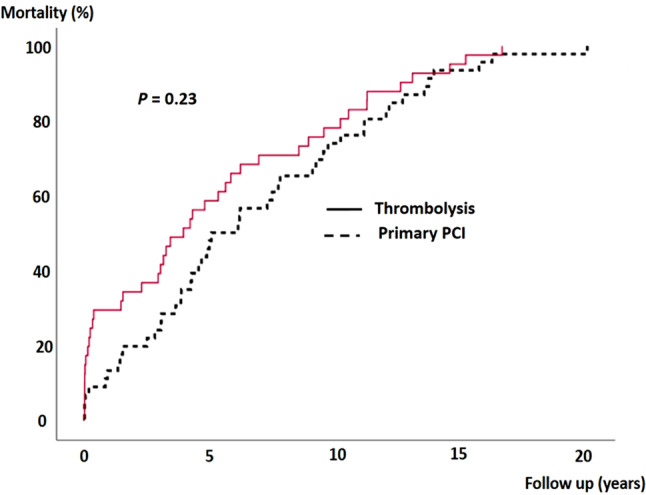
Mortality of a total of 87 patients > 75 years with ST-elevation myocardial infarction randomised to either primary percutaneous coronary intervention (*PCI*) (*n* = 46) or thrombolysis (*n* = 41)

By January 2021, all the patients had died. Mean survival was 6.0 years (SD 5.0). Maximum survival was 20 years. The patients randomised to streptokinase died after a mean follow-up duration of 5.2 years (SD 4.9) compared to 6.7 years (SD 5.0) in patients randomised to primary PCI (*p* = 0.15). Thus, the mean final survival benefit of primary PCI was 1.5 years. Long-term survival is depicted in Fig. 1.

The life expectancy of the general Dutch population, adjusted for year of inclusion, age and gender was 7.65 years, demonstrating that the life expectancy of patients treated with thrombolysis was 2.45 years and of the patients treated with primary PCI 0.95 years shorter as compared to the general population.

## Discussion

We demonstrate the long-term sustained benefit of primary PCI as compared to thrombolysis in STEMI patients > 75 years of age. Our results are in line with long-term data on younger patients [6]. Although life expectancy in both treatment groups was shorter compared to the general Dutch population, survival after primary PCI was substantially better than after thrombolytic therapy.

The benefit of primary PCI as compared to thrombolysis after a short follow-up has been demonstrated in both randomised [3–5] and observational studies [7]. It has even been suggested that primary PCI may become more beneficial with increasing age [8]. Not surprisingly, real-world data on patients with STEMI who are not treated with primary PCI show that the oldest patients have the worst prognosis [9]. Also in a real-world setting, it was shown that in nonagenarian STEMI patients primary PCI can be performed with good clinical outcomes and acceptable in-hospital mortality rate and must be recommended irrespective of their age, even in very old patients [10].

Since the benefit of primary PCI in older patients with STEMI has been demonstrated in both randomised and observational studies, also in those with a serious comorbidity such as dementia [11], there should be discussion as to which subgroups of older patients should not be treated invasively. These subgroups may include patients with either a serious comorbidity such as severe dementia or those who present with a very poor cardiac prognosis (such as those with a very high age and cardiogenic shock). It is remarkable that there are many papers about the treatment of myocardial infarction, but almost no papers on treatment restriction in myocardial infarction, including restriction of life-sustaining treatment and do-not-resuscitate orders.

Our study has several limitations. We had neither data on quality of life nor on recurrent myocardial infarction or the need for revascularisation during long-term follow-up. Also, data on cardiac or non-cardiac death are lacking, as well as data on frailty or quality of life. Furthermore, the number of patients in of our study was small, and as the study was halted prematurely, the anticipated sample size could not be reached. This was also an issue in the Senior PAMI study. As in all studies with a very long follow-up, both invasive and non-invasive treatment have improved since the time of randomisation. Finally, our findings should be interpreted under consideration of the fact that particularly older patients included in clinical trials may differ from those in a real-world setting.

In conclusion, in older patients with STEMI primary PCI may potentially prolong life expectancy by approximately 1.5 years as compared to thrombolysis.
